# Supplementary material for: Survival-Related Genes on Chromosomes 6 and 17 in Medulloblastoma
Source: Int J Mol Sci. 2024 Jul 9;25(14):7506. doi: 10.3390/ijms25147506 (PMC11277021; doi:10.3390/ijms25147506)
Supplement: Supplementary file 1 [file ijms-25-07506-s001.zip › supplementary figures.pdf]

A.

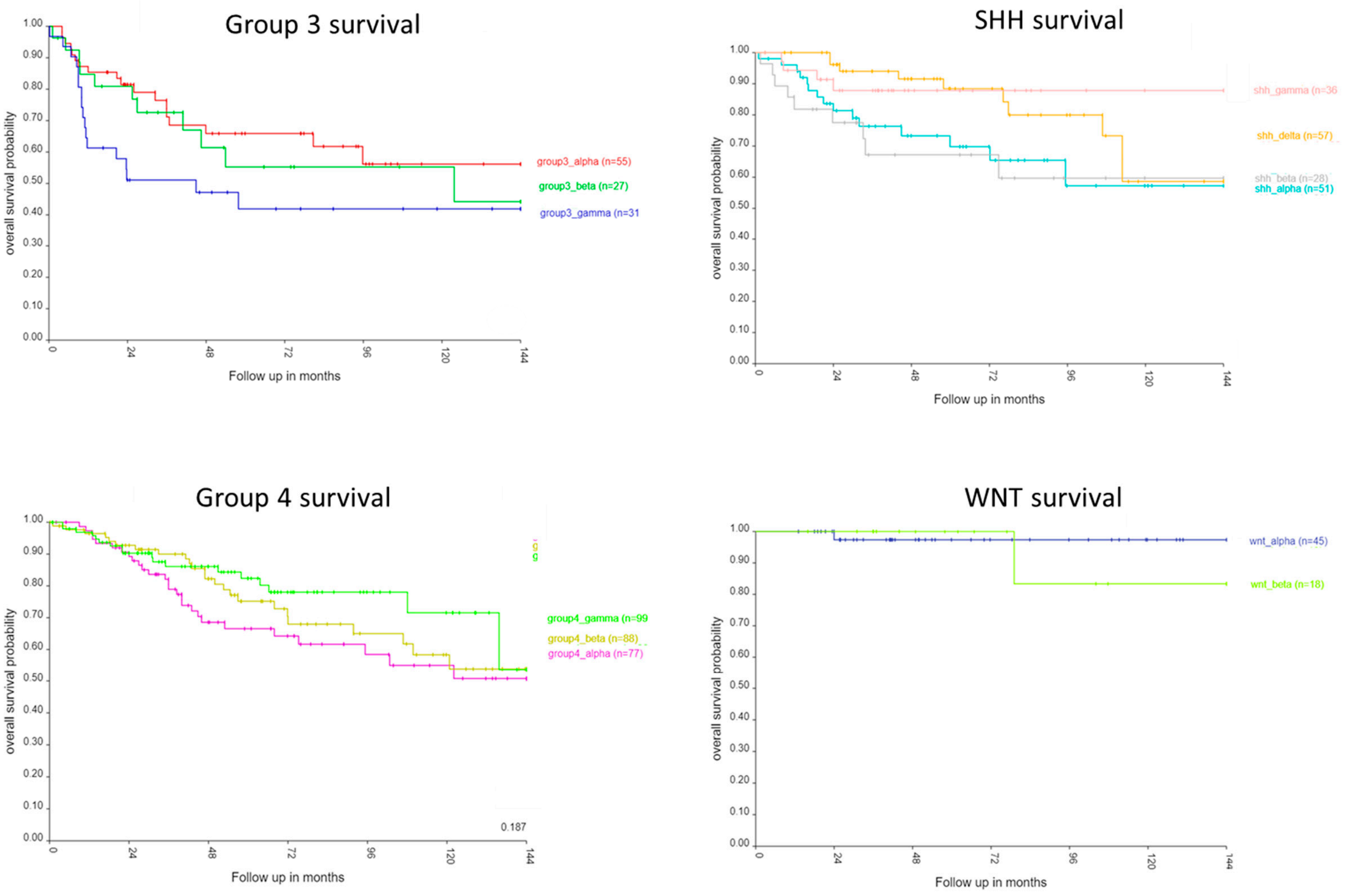

Group 3 survival
overall survival probability
Follow up in months
group3_alpha (n=55)
group3_beta (n=27)
group3_gamma (n=31
SHH survival
overall survival probability
Follow up in months
shh_gamma (n=36
shh_delta (n=57)
shh_beta (n=28)
shh_alpha (n=51)
Group 4 survival
overall survival probability
Follow up in months
group4_gamma (n=99
group4_beta (n=88)
group4_alpha (n=77)
0.187
WNT survival
overall survival probability
Follow up in months
wnt_alpha (n=45)
wnt_beta (n=18)

B.

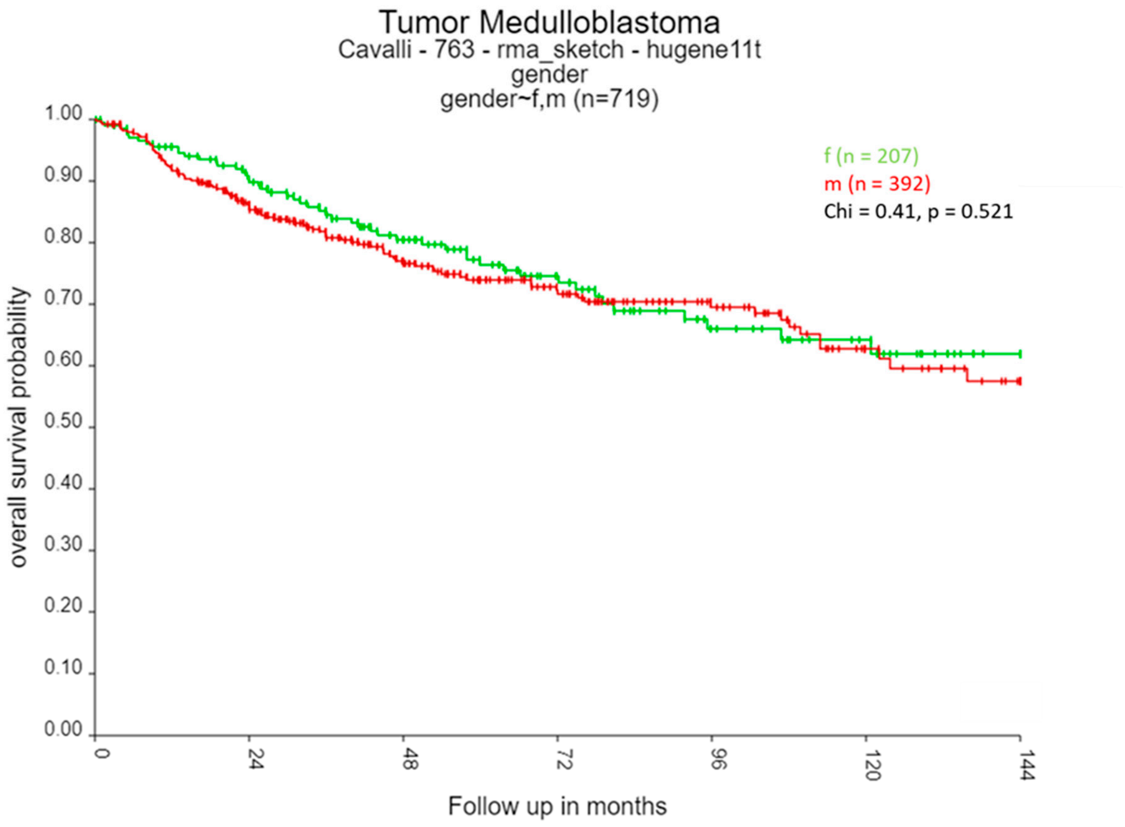
Tumor Medulloblastoma
Cavalli - 763 - rma_sketch - hugene11t
gender
gender~f,m (n=719)
f (n = 207)
m (n = 392)
Chi = 0.41, p = 0.521
overall survival probability
1.00
0.90
0.80
0.70
0.60
0.50
0.40
0.30
0.20
0.10
0.00
0
24
48
72
96
120
144
Follow up in months

Supplemental Figure 1. Kaplan-Meier survival curves for MB subtypes and gender. A. KM curves for MB subtypes. B. KM curves by gender.

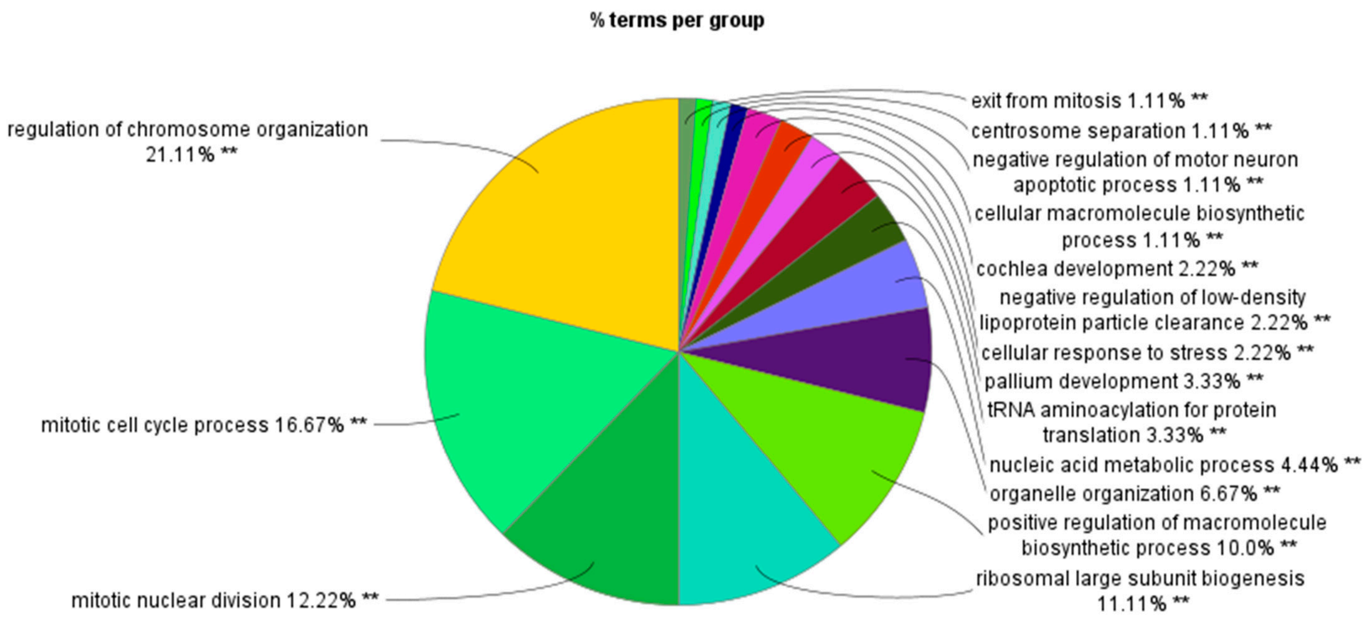

Supplemental Figure 2. Percentage of GO (Gene Ontology) terms per group for all SRGs
